# Linen Leagues for Hospitals: Their Growing Activities and Value

**Published:** 1916-03-25

**Authors:** 


### THE MATRONS' AND SISTERS' DEPARTMENT.

### LINEN LEAGUES FOR HOSPITALS.

### Their Growing Activities and Value.

In our issue of February 19 we recorded that the Royal Devon and Exeter Hospital Linen League is, despite the war, increasing its work in proportion to the needs of the patients. It is interesting to compare Miss Small's account of the work done for the Exeter Hospital with that of the newly formed Linen League of the South Devon and East Cornwall Hospital at Plymouth. While the Exeter Hospital has been provided by its League with all the linen used for the patients during the last eleven years, the League at Plymouth is now celebrating its first anniversary. Last year the Exeter League supplied 1,448 articles in addition to subscriptions to the value of £129, while the South Devon and East Cornwall League contributed 2,271 articles. We hope that the Plymouth League will maintain its contributions and be able to show as good a record of work in years to come as has been the case at Exeter.

At Preston the energy put into the Linen League first of all supplied all the money necessary for the whole of the linen required in the wards of the Royal Infirmary. Then the Linen League took on in addition the supply of linen for the hospital household, and during the last few years it has taken in hand other plans to yield comfort to the patients. In one year easy-chairs were voted to be desirable in the wards, and so through the Linen League they were selected, supplied, and paid for. Another year an enterprising sister, no doubt, pointed out that the popular balcony devoted to female patients would be infinitely more comfortable and better equipped if it had an awning. With businesslike aptitude the cost was fixed at £50, and this £50 was speedily provided. We have no doubt that up and down the country these facts will prove of interest to the workers for, and supporters of, many a hospital. Then the Preston plan in a few years should prevail in many centres of population, when similar comforts for hospital patients in exact proportion to the public spirit and alertness which characterise the inhabitants will be provided for their voluntary hospitals.

At the annual meeting of the Plymouth Linen League just referred to, short speeches made by the Matron and Mrs. Grant, Hon. Secretary and Treasurer, referred to the need for a hospital laundry. Mrs. Grant stated that 4,000 articles were sent to wash weekly at a cost last year of £738, and it was hoped that the initial cost of a laundry would soon be repaid. Miss Hopkins, matron of the hospital, said she knew, after thirty years of office, how to be grateful for the establishment of this League. She thought that no one but a matron could fully appreciate what a good supply of linen meant. She supported Mrs. Grant in her suggestion that a hospital laundry was much needed. She hoped that the suggestion would take immediate effect, because she could not bear to think of all the beautiful things they had been given being torn to shreds in a few months. We heartily support Mrs. Grant and Miss Hopkins in their struggle for a well-equipped steam laundry, and are sure that it would prove an economy in the wear and tear of linen and in expense, besides being a most instructive addition to the hospital organisation.

The developments in regard to the Linen League at the Royal Infirmary, Preston—which is far too little known to hospital people, though it is one of the most interesting voluntary hospitals to visit—are important. The voluntary system has proved over and over again that the one thing needful to secure a revenue for a successful voluntary hospital is to extend the area of interest in it to the fullest possible extent. The old idea of letting a few families take charge of the hospital affairs of a provincial town and devote themselves to its interests had merits, but it only sufficed to meet the needs of the community in the days when hospitals were relatively small and relatively dreaded institutions. Nowadays it is the small giver who is sought out, for once he or she is won the interest is continuous, and as the power of giving in such supporters is necessarily limited the best of them often expend much energy in recruiting friends to join the personal service army which brings grist to the hospital mill.

### Round the Hospitals.

